# Gastrointestinal Bleeding Secondary to Portal Hypertensive Duodenopathy in a Patient with Decompensated Liver Cirrhosis

**DOI:** 10.1155/2018/9430701

**Published:** 2018-10-18

**Authors:** Rohini Krishna, Samuel O. Igbinedion, Richie Diaz, Nazneen Hussain, Moheb Boktor

**Affiliations:** ^1^Department of Internal Medicine, Louisiana State University Health Sciences Center, Shreveport, LA, USA; ^2^School of Medicine, Louisiana State University Health Sciences Center, Shreveport, LA, USA; ^3^Department of Gastroenterology and Hepatology, Louisiana State University Health Sciences Center, Shreveport, LA, USA

## Abstract

With alcoholic cirrhosis and nonalcoholic fatty liver disease continuously on the rise in the United States, there is also a corresponding rise in portal hypertension. Portal hypertensive duodenopathy (PHD) is a complication of portal hypertension not commonly seen in cirrhotic patients. We present a case of a 46-year-old man who presented with decompensated liver cirrhosis secondary to gastrointestinal bleed. The patient underwent esophagogastroduodenoscopy (EGD) with findings indicative of PHD. Patient subsequently underwent transjugular intrahepatic portosystemic shunt (TIPS) with resolution of gastrointestinal bleed. We highlight TIPS as a management strategy in patients with PHD for whom less invasive measures are not effective.

## 1. Introduction

Portal hypertension is a common complication of chronic liver disease and is primary responsible for most clinical presentations of cirrhosis [1]. In the United States, portal hypertension often occurs secondary to cirrhosis. Noncirrhotic portal hypertension accounts for a minor number of cases [1]. Known complications of portal hypertension include esophageal varices, ascites, portal hypertensive gastropathy, spontaneous bacterial peritonitis, and hepatorenal syndrome, among many others [1]. Portal hypertensive duodenopathy (PHD) is a less common finding of portal hypertension syndrome in cirrhotic patients [2]. It is estimated to occur in 14 to 25% of cirrhotic patients [3]. A recent European retrospective study estimates its prevalence to be 8.4% when duodenal erosions/ulcers are excluded from the definition, due to the inability to rule out a peptic origin in a majority of the patients [2].

Defining features of PHD on endoscopy include erythema, scattered petechiae, friable mucosa, erosions, ulcers, edema, lesions, and duodenal polyps. There have been several reports detailing these varying endoscopic findings in the duodenum consistent with PHD [3–6]. These findings can be responsible for overt or occult signs of gastrointestinal bleeding and thus need to be highlighted for clinical benefit [7]. We report a case of gastrointestinal bleed secondary to portal hypertensive duodenopathy in a patient with decompensated liver cirrhosis. We intend to highlight PHD as a cause of acute gastrointestinal bleed in cirrhotic patients.

## 2. Case Report

A 46-year-old male with a history of alcoholic liver cirrhosis complicated by small esophageal varices after banding and moderate ascites was awaiting liver transplant (MELD 24, Child-Pugh class B). He presented with a 3-day history of abdominal pain. He described the pain as sharp and located around the periumbilical area with notable suprapubic discomfort. He also reported associated symptoms of nausea, hematochezia, and general malaise. On further review of systems, he reported chills, increased fatigue, shortness of breath, lightheadedness, and decreased appetite 4 days prior to presentation. He reported compliance at home with a sodium-restricted diet and medications. Physical exam was significant for abdominal distension with positive fluid wave, generalized abdominal tenderness, and splenomegaly. Scant blood was noted on the rectal exam. Laboratory studies revealed stable hemoglobin of 12.4 g/dL, hematocrit of 36.8%, platelet of 83 K/UL, leukocytosis of 15.4 K/UL with 78% neutrophils and 16% bands, sodium of 130 mmol/L, ammonia of 83 Umol/L, ALT of 40 U/L, AST of 42 U/L, and alkaline phosphatase of 127 U/L. International normalized ratio (INR) was 2.02. Diagnostic paracentesis revealed serosanguinous fluid and ascitic fluid polymorphonuclear neutrophils (PMN) count of 686 cells/mm^3^, consistent with culture negative neutrocytic ascites. Blood cultures revealed no growth of organisms. The patient was started on intravenous (IV) ceftriaxone, IV pantoprazole infusion, and an IV bolus of octreotide followed by continuous infusion. He completed a 5-day course of IV ceftriaxone therapy.

**Figure 1:**
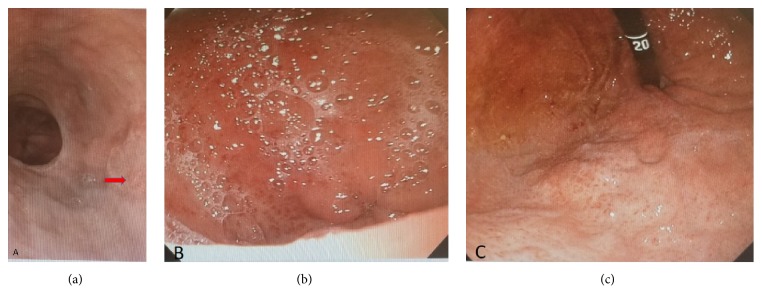
Endoscopy findings of ulcerations in the distal esophagus (arrow) from prior banding of small esophageal varices and diffuse portal hypertensive gastropathy.

**Figure 2:**
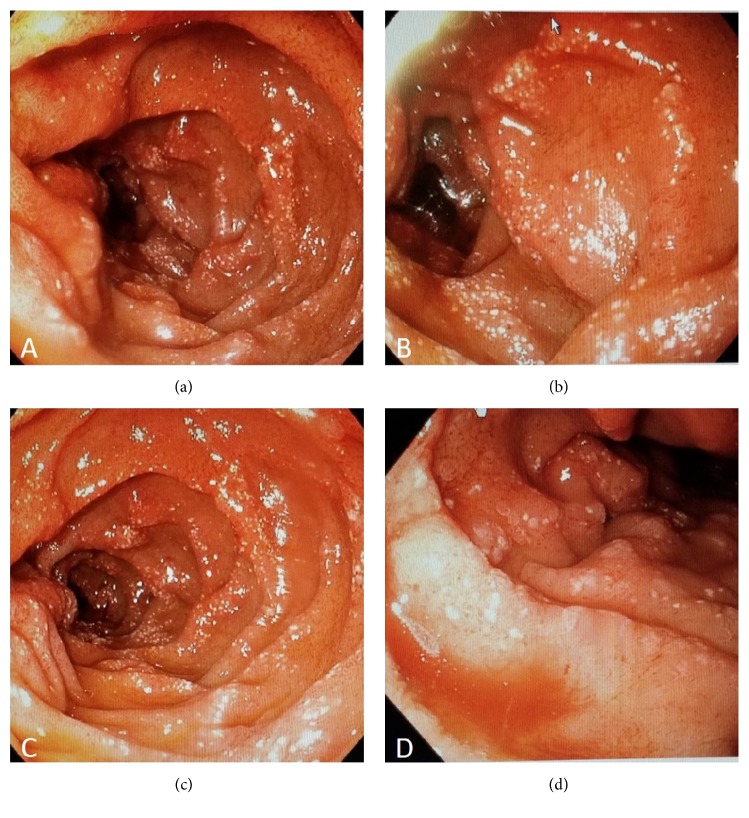
Endoscopic findings of erosions, mucosal erythema, and edema in the duodenum with minor oozing of blood noted.

The patient underwent an esophagogastroduodenoscopy (EGD) which showed ulcerations in the distal esophagus from prior banding of small esophageal varices and diffuse portal hypertensive gastropathy (Figure 1). Mucosal edema and erythema with an area of oozing of blood were identified in the duodenum bulb and between proximally located duodenal folds with no specific identifiable lesion. These findings correlated with a diagnosis of portal hypertensive duodenopathy (Figure 2). Colonoscopy was performed, and it showed no active bleeding. However, there were old blood clots in the terminal ileum in addition to mild inflammation in the terminal ileum near the ileocecal valve. Video capsule endoscopy was also performed, and it revealed no active bleeding. Because of the concern of the possible failure of the patient on medical therapy for portal hypertension while awaiting liver transplant, the patient was referred for evaluation by a transjugular intrahepatic portosystemic shunt (TIPS). The patient successfully underwent the TIPS procedure. Follow-up occurred one month later in clinic. At this time, the patient reported resolution of the hematochezia.

## 3. Discussion

Portal hypertensive duodenopathy is a clinical condition that describes the mucosal and histopathologic abnormalities in the duodenum in patients with portal hypertension [7, 8]. Some reports consider this condition in a different category, portal hypertensive enteropathy [8]. Mucosal erythema, mucosal edema, mucosal breaks (erosions or ulcers), and vascular lesions are findings that characterize PHD as a condition [7]. Duodenal polyps have been identified in newer reports as another finding secondary to PHD [5, 6]. These findings are only observed and categorized under patient evaluation with EGD, video capsule endoscopy, or deep enteroscopy [8]. Clinical manifestation of these findings can range from asymptomatic presentation to fatal gastrointestinal bleeding [8]. In a 2007 descriptive study on portal hypertensive patients by Barakat et al., 51.4% were found to have endoscopic features of PHD, with PHD identified as the source of GI bleed in close to 10% of the patients [7]. Histopathologic evidence of duodenopathy, including intestinal vasculopathy, was prevalent in 85% of the patients, with no difference in prevalence between patients with and without endoscopic duodenopathy [7]. This highlighted that the histopathologic evidence of duodenopathy does not always indicate endoscopic duodenopathy, hence lending no clinical utility.

Our patient presented with endoscopic features concerning PHD. The patient also had evidence of diffuse portal hypertensive gastropathy on endoscopy. Predisposing risk factors for PHD are difficult to pinpoint. On analysis of PHD versus other endoscopic findings in patients with portal hypertension, Barakat et al. (2007) revealed the absence of correlation between PHD and the size of esophageal varices and/or presence of variceal bleed. There was, however, a relation to the extent and severity of gastric lesions [7]. Further studies seem to suggest a relationship between PHD development and the degree of portal hypertension as demonstrated by large esophageal varices, severe portal hypertensive gastropathy, and high hepatic venous pressure gradients [2, 9]. However, other reports have diminished its relationship to the portal pressure, instead identifying the point at which the pressure causes congestive changes to be the culprit in PHD development [7, 10].

The association of PHD in patients with prior variceal band ligation is very significant, and this relationship is well documented [2, 11]. One such study showed an increase in the number of patients with PHD from 6.6% to 46.7% after variceal band ligation (p < 0.001) [12]. This study linked the development of PHD with an increase in VEGF expression in gastric and intestinal cells after variceal obliteration [12]. Our patient had other evidence of portal hypertension prior to development of PHD, but the history of prior endoscopic band ligation was significant in his development of PHD. He was found to have PHD on subsequent endoscopy with evidence of overt GI bleed. Our case highlights the importance of identifying PHD in patients with decompensated liver cirrhosis, as this could possibly lead to a devastating gastrointestinal bleed.

Treatment of PHD is not established, and recommendations are based on strategies documented in case reports [5, 11, 13]. Medical therapy which includes prophylaxis with beta blocker has been notably effective [13]. Endoscopic therapy, which includes polypectomy, endoclip use, and argon plasma coagulation, has been reported to be successful [8, 13]. However, liver transplant is the optimal treatment strategy in patients who are surgical candidates, and PHD resolves in these patients afterwards [2]. This is likely due to the resolution of the portal hypertension syndrome. In some cases like this reported, PHD can be the cause of either self-limited bleeding and erythematous lesions or even massive hemorrhage requiring emergent shunting procedures [7]. We report a rare case of gastrointestinal bleeding secondary to PHD. Further prospective studies are needed to better understand the pathophysiology of PHD. Clinicians should also consider TIPS as a management strategy in patients with PHD for whom less invasive measures are not effective.
